# The evolution of comorbidities in chronic diseases among Chinese middle-aged and elderly people: Evidence from the CHARLS (2015-2020)

**DOI:** 10.1371/journal.pone.0329372

**Published:** 2025-08-04

**Authors:** Zihui Zhang, Chuhui Hu, Yufeng Cai, Fei Liu, Yongheng Duan, Xusheng Wu, Dehua Hu

**Affiliations:** 1 Department of Biomedical Informatics, School of Life Sciences, Central South University, Changsha, Hunan, China; 2 Shenzhen Health Development Research and Data Management Center, Shenzhen Guangdong, China; University of Illinois Urbana-Champaign, UNITED STATES OF AMERICA

## Abstract

**Background:**

The comorbidity of chronic diseases among middle-aged and elderly people is a global public health concern that has attracted great attention in recent years. It is crucial to explore the evolutionary pattern of chronic disease comorbidity in Chinese middle-aged and elderly people and to reveal the developmental trajectory of chronic diseases in this population.

**Methods:**

Data from the China Health and Retirement Longitudinal Study (CHARLS 2015–2020) were utilized for the fixed cohort analysis. Based on the prevalence information of 14 chronic diseases (including hypertension, dyslipidemia, diabetes, cancer, chronic lung diseases, liver disease, heart disease, stroke, kidney disease, stomach diseases, emotional problems, memory-related diseases, arthritis, and asthma) among 10,089 participants aged ≥45 years, association rules and cluster analysis were used to identify trends and trajectories of comorbidities in the middle-aged and elderly populations in China.

**Results:**

The analysis revealed that the comorbidity rate of the 14 chronic diseases showed a consistent annual increase from 2015–2020. By 2020, over 85% of patients diagnosed with a single chronic condition exhibited concurrent multimorbidity. This epidemiological progression was paralleled by a progressive increase in detected disease associations: binary comorbidities rose from three significant associations in 2015–10 in 2020, whereas higher-order combinations expanded from one ternary association in 2015–35 ternary and 18 quaternary associations by 2020. Notably, hypertension maintained a central position across all identified comorbidity clusters. The comorbidity patterns identified in 2015 included respiratory, liver and kidney, and cardio-cerebral comorbidity patterns and cancer and emotional problems. The comorbidity patterns identified in 2018 included respiratory, liver and kidney, cerebrovascular, and cardiovascular metabolic comorbidity patterns. The comorbidity pattern in 2020 was the same as that in 2018.

**Conclusion:**

The issues of comorbidities in chronic diseases among Chinese middle-aged and elderly people is significant, with observed variations in the comorbidity patterns across different time periods. The development of clinical assessment and management guidelines for chronic diseases comorbid with key conditions, such as hypertension and dyslipidemia, is recommended. These guidelines aim to facilitate the co-management, co-treatment, and co-reduction of multiple diseases among middle-aged and elderly people.

## 1 Introduction

With significant advancements in medical and health services, life expectancy has increased substantially. Comorbidity has become a prevalent issue among elderly patients with chronic diseases, emerging as a critical threat to human health and survival. According to the Expansion of Morbidity Hypothesis [1], medical technological progress, while extending life expectancy, also prolongs the course of chronic disease and leads to the superimposition of multiple health conditions. Diseases that were once fatal have been transformed into manageable long-term conditions through effective interventions, creating temporal windows for additional chronic diseases to develop. As outlined in the Healthy China Initiative (2019–2030) [2], over 180 million people aged ≥60 years in China suffer from chronic diseases, approximately 50% of whom exhibit comorbidity patterns. Chronic disease comorbidity refers to the coexistence of two or more non-infectious chronic diseases in a single patient [3]. The senescence of tissues and organs and the decline in physiological functions due to advancing age lead to increased susceptibility to comorbidities at the organismal level. Meanwhile, the presence of comorbidities not only impacts patients’ daily functioning but may also progressively lead to psychosomatic disorders, potentially evolving further into cognitive-affective psychosomatic conditions [4–6]. However, the current landscape demonstrates a notable downward shift in the age of chronic disease onset [7], with middle-aged populations experiencing rising chronic disease prevalence, thereby representing an emerging public health challenge. This trend of premature chronic disease manifestation underscores the scientific necessity of integrating middle-aged cohorts into comorbidity research frameworks. Such inclusion facilitates a more systematic understanding of chronic disease trajectories across the lifespan and provides a strong empirical foundation for developing targeted public health strategies.

Currently, China has entered a phase of deep aging, with chronic diseases increasingly affecting middle-aged and elderly populations [8,9]. Chronic disease comorbidities have become a major cause of patient disability, increased mortality, decreased quality of life, and increased utilization rate of medical services. Moreover, this phenomenon has led to increasing financial burdens and escalating social pressure annually. The Chinese government places significant emphasis on addressing chronic disease comorbidity. The Tutorial for Outline of the Healthy China 2030 Plan [10], released in 2016, explicitly proposes a comprehensive strategy for the prevention and control of chronic diseases. Thereafter, China’s Medium-to-Long Term Plan for the Prevention and Treatment of Chronic Diseases (2017–2025) [11], released in 2017, emphasized expanding the prevention and treatment of chronic diseases, reducing the burden of diseases on society and individuals, and enhancing the healthy life expectancy of residents. Furthermore, in the Healthy China Action (2019–2030) released in 2019, 15 special actions were further refined to comprehensively and thoroughly implement the contents of the plan.

Chronic disease comorbidities are widespread and diverse, making it particularly challenging to assess their prevalence rate and overall impact on the general population [12,13]. However, failing to accurately and promptly assess comorbidities can lead to challenges in diagnosing and treating patients, complicating medical decision-making and increasing uncertainty in risk assessments. Therefore, investigating the progression of chronic disease comorbidities is vital for effectively preventing and managing these conditions in China and worldwide. Currently, the CHARLS database has been extensively utilized by numerous researchers in this field. For instance, Hu et al. [14] used data from the CHARLS database from 2011–2018 to develop and verify a weighted index of China’s morbidity rate for middle-aged and elderly people in the community, effectively quantifying the burden of morbidity and comorbidity in these populations and facilitating community health management. Zhang et al. [15] and Fan et al. [16] used data from the CHARLS database (2018) to identify comorbidity patterns and related risk factors through latent category analysis, which is important for the selection of preventive interventions and treatment strategies. Guo et al. [17] used data from the CHARLS database in 2015 and adopted the Apriori algorithm to explore the spatial distribution and pattern of morbidity among elderly individuals in China. However, most of these studies rely on cross-sectional research methods, while some longitudinal studies struggle to maintain stable research subjects and lack continuous observations of disease progression within the same group over time. Consequently, they are unable to capture the dynamic progression of diseases over time and have limitations in investigating comorbidities in chronic diseases.

To address these limitations, this study utilized data from the CHARLS database (2015–2020) to assess temporal changes in comorbidity trends. Using cluster analysis and association rule mining algorithms, this study systematically investigated the progression patterns of chronic disease comorbidities in China’s middle-aged and elderly population. The cluster analysis methodology enables data categorization by grouping individuals with similar clinical profiles into distinct clusters. Concurrently, association rule analysis identifies statistically significant disease co-occurrence patterns by examining inter-item relationships within multidimensional health datasets. Together, these methods create a robust analytical framework for understanding the developmental trajectories and interaction mechanisms of multimorbidity in this demographic. These findings will enhance our understanding of morbidity development in middle-aged and older patients, provide novel insights into their underlying mechanisms or etiology, and further strengthen self-management strategies and preventive measures for individuals with chronic conditions.

## 2 Methods

### 2.1 Study design and participants

The data for this study was obtained from the database of CHARLS, which is a nationally representative longitudinal survey of persons in China aged ≥45 years and their spouses. Informed consent was obtained from all participants. The study protocol was approved by the Ethics Review Committee of Peking University (IRB00001052–11015). Follow-up surveys, with publicly available data considered here, were conducted in 2015 (wave 3), 2018 (wave 4), and 2020 (wave 5).

The sample of participants in this study followed the following inclusion and exclusion criteria. The inclusion criteria were as follows: (1) participated in all three survey waves and (2) aged ≥45 years at the 2015 baseline survey. Meanwhile, the exclusion criteria were as follows: (1) inconsistent or missing records of three demographic variables (e.g., age, sex); and (2) missing critical data or entries with logical inconsistencies (e.g., discrepancies in disease response records).

**Fig 1:**
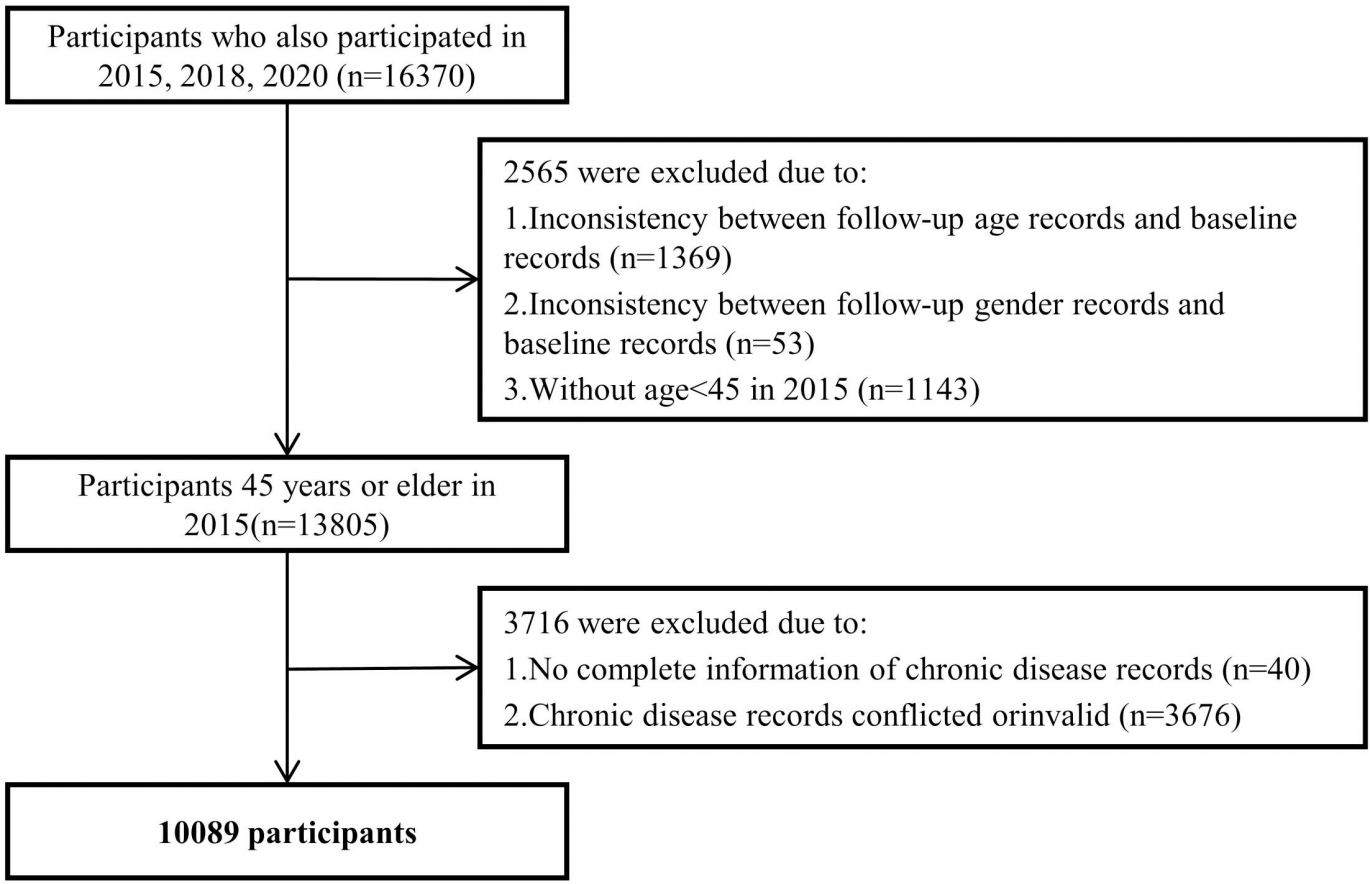
Sample screening process.

After excluding participants with incorrect records of demographic variables and those with missing records and logical conflicts, 10,089 valid samples were ultimately included. The sample screening logic is shown in Fig 1.

### 2.2 Assessment of chronic diseases and comorbidity

Chronic disease prevalence was determined using standardized self-report measures, specifically participant responses to physician-diagnosed conditions through validated survey items such as, “Did the doctor tell you that you have the following chronic diseases?” and “Do you know that you have the following chronic diseases?” Each survey wave systematically collected diagnostic confirmation and disease progression data (including both diagnosis dates and symptom onset) for 14 chronic diseases: hypertension, dyslipidemia, diabetes, cancer, chronic lung diseases, liver disease, heart disease, stroke, kidney disease, stomach diseases, emotional problems, memory-related diseases, arthritis, and asthma. In this study, Parkinson’s disease was classified as a memory-related disease, and all site-specific malignancies were classified as cancer (Table 1).

**Table 1:** Questionnaire data extraction range.

Code (Year)	Question	Notes
**ZDA007 (2015)**	[Chronic Disease] reported by participants during the last wave interview.	
**DA007 (2015)**	Have you been diagnosed with [Chronic Disease] by a doctor?	
**DA007_W2_1 (2015)**	Have you been diagnosed with [Chronic Disease] by a doctor [in the last two years]?	If the answer is “2. Disagree”, then correct ZDA007.
**DA007_W2_2 (2015)**	Since your last visit (two years ago), has any doctor ever told you that you have any of the following [Chronic Disease]?	
**DA008 (2015)**	Do you know if you have [Self-known Chronic Disease]?	The answer “1. Yes, I know I have it” is considered as having the disease.
**Zdisease (2018)**	[Chronic Diseases] reported by participants during the last wave interview.	
**ZDA008 (2018)**	[Self-known Chronic Disease] reported by participants during the last wave interview.	
**DA010_W2_2 (2018)**	Doctor diagnosed [Chronic Disease] compared to the last wave interview.	If the record indicates that the participant had the disease in 2015 but the answer is “99 Never Had the Disease”, then delete this record.
**DA007 (2018)**	Have you been diagnosed with [Chronic Disease] by a doctor?	
**DA008 (2018)**	Do you know if you have [Self-known Chronic Disease]?	
**Zdisease (2020)**	[Chronic Disease] reported by participants during the last wave interview.	
**Zselfdisease (2020)**	[Self-known Chronic Disease] reported by participants during the last wave interview.	
**DA002 (2020)**	Doctor diagnosed [Chronic Disease] compared to the last wave interview.	If the record indicates that the participant had the disease in 2018 but the answer is “99 Never Had the Disease”, then delete this record.
**DA002_1 (2020)**	[Self-know Chronic Disease] compared to the last wave interview.	If the record indicates that the participant had the disease in 2018 but the answer is “99 Never Had the Disease”, then delete this record.
**DA003 (2020)**	Have you been diagnosed with [Chronic Disease] by a doctor?	
**DA004 (2020)**	Do you know if you have [Self-known Chronic Disease]?	

### 2.3 Variable selection and definition

Data extraction from the CHARLS database focused on two primary domains: demographic characteristics and health status/functional capacity. Demographic characteristics (sex, age, marital status, and education level) of valid samples were extracted in this study. Health status/functional capacity includes the prevalence data for 14 chronic diseases. The definitions and descriptions of the specific variables are detailed in Table 2.

**Table 2:** Meaning of main variables.

Variable name	Variable definition
**Sex**	Based on the item, “Interviewer record the Respondent’s sex” Male = 1 Female = 2
**Age**	The age group of the participants corresponds to the classification standard for elderly individuals proposed by the World Health Organization. 45–59 = 1 60–74 = 2 ≥75 = 3
**Marital status**	Based on the item, “What is your marital status?” and “Do you have a mate living with you as a couple (cohabit)?” Married with Spouse Present = 1 Married But Not Living with Spouse Temporarily for Reasons Such as Work = 2 Separated = 3 Divorced = 4 Widowed = 5 Never Married = 6 Nonmarital Cohabitation = 7
**Education level**	0 year = 1 (including no formal education) 1–6 years = 2 (including did not finish primary school, sishu/home school and elementary school) 7 years or higher = 3 (including middle school, high school, vocational school, two-/three-year college/associate degree, four-year college/bachelor’s degree, master’s degree and doctoral degree/Ph.D.)
**Chronic disease**	Chronic disease includes hypertension, dyslipidemia, diabetes, cancer, chronic lung diseases, liver disease, heart disease, stroke, kidney disease, stomach diseases, emotional problems, memory-related diseases, arthritis, and asthma. Have chronic disease[i]=1 No chronic disease[i]=0
**Prevalence**	Chronic disease prevalence was ascertained through standardized self-report measures, specifically participant responses to physician-diagnosed conditions via validated survey items. No disease = 1 1 disease = 2 Comorbidity = 3

### 2.4 Statistical analysis

The basic information of the participants was described by frequency and percentage, and the prevalence of chronic diseases and comorbidities was reported as percentage and mean ± standard deviation (SD). The prevalence rate is calculated as the number of individuals diagnosed with the disease divided by the total population, multiplied by 100%. For association rule identification across chronic disease data from 2015–2020, we implemented the Apriori algorithm using R version 4.3.3, with subsequent visualization of association rules through specialized plotting functions. Cluster analysis was performed using hierarchical clustering with a custom Yule’s Q distance metric. All analytical workflows, including distance matrix computation, cluster dendrogram generation, and cluster statistics reporting, were programmatically executed within the R environment.

## 3 Results

### 3.1 Basic information

Table 3 shows the baseline characteristics of the study sample. A total of 10,089 participants were included in this study, of which 49.08% were male and 50.92% were female. The age group of the participants corresponds to the classification standard for elderly individuals proposed by the World Health Organization. However, the sample size of the longevous individuals in the retained samples is very small. In 2015, this subgroup consisted of only five individuals, leading to their inclusion in the older age group for statistical convenience. The marriage status grouping follows the original grouping of the CHARLS database, which consists of seven distinct categories. Furthermore, for participants who responded “Yes” to the question “Do you have a partner living together as a spouse” but are unmarried, this study considers their marital status to be cohabitation without marriage. According to the education level data, 21.6% of the participants had never received education, 43.6% had 1–6 years of education, and 34.8% had > 6 years of education. In terms of disease conditions, the middle-aged and elderly people with chronic disease comorbidities accounted for 34.3% and the number of participants without chronic disease accounted for 36.4% in 2015. In 2020, participants with chronic disease comorbidities accounted for 59.9%, whereas those without chronic disease accounted for only 17.7%.

**Table 3:** Participants’ demographics from 2015–2020.

		2015	2018	2020
**Sex, n (%)**	Male	4952 (49.08%)	4952 (49.08%)	4952 (49.08%)
	Female	5137 (50.92%)	5137 (50.92%)	5137 (50.92%)
**Age, n (%)**	45–59 (The Middle-Aged)	6165 (61.1%)	4926 (48.8%)	4208 (41.7%)
	60–74 (The Young Old)	3448 (34.2%)	4379 (43.4%)	4811 (47.7%)
	75–89 (The Old Old)	476 (4.7%)	784 (7.8%)	1070 (10.6%)
**Marital status, n (%)**	Married with Spouse Present	8413 (83.4%)	8105 (80.3%)	7760 (76.7%)
	Married But Not Living with Spouse Temporarily for Reasons Such as Work	570 (5.6%)	609 (6.1%)	763 (7.6%)
	Separated	28 (0.3%)	31 (0.3%)	37 (0.4%)
	Divorced	68 (0.7%)	80 (0.8%)	80 (0.8%)
	Widowed	943 (9.3%)	1211 (12.0%)	1392 (13.8%)
	Never Married	59 (0.6%)	51 (0.5%)	53 (0.5%)
	Nonmarital Cohabitation	8 (0.1%)	2 (0.0%)	7 (0.1%)
**Education level, n (%)**	0 years	2183 (21.6%)	2183 (21.6%)	2183 (21.6%)
	1–6 years	4399 (43.6%)	4399 (43.6%)	4399 (43.6%)
	7 years or higher	3507 (34.8%)	3507 (34.8%)	3507 (34.8%)
**Prevalence**	No disease	3668 (36.4%)	2489 (24.7%)	1789 (17.7%)
	1 disease	2957 (29.3%)	2651 (26.3%)	2255 (22.4%)
	comorbidity	3464 (34.3%)	4949 (49.0%)	6045 (59.9%)

### 3.2 Chronic diseases prevalence and comorbidity

Table 4 summarizes the number of patients with different number of diseases. The comorbidity rates of 14 chronic diseases have shown an increasing trend annually from 2015–2020, and all of them exceeded 85% by 2020. In 2020, the prevalence of hypertension and arthritis was higher at 42.6% and 40.9%, respectively, indicating that approximately half of the participants had these two chronic diseases. Additionally, the average number of comorbidities exhibited a consistent upward trend over the years, with significant increases in cancer and emotional problems, rising by approximately 60% and 52%, respectively. The substantial increase in the comorbidity rate reflects the intricate interplay and associations among diverse chronic diseases, which may encompass lifestyle choices, genetic predispositions, environmental factors, and treatment modalities. Therefore, further investigation is warranted to elucidate the intricate relationship between these factors and to implement appropriate preventive measures and therapeutic strategies. Furthermore, diseases with low incidence tend to exhibit a higher average number of comorbidities compared to those with high incidence.

**Table 4:** The prevalence and comorbidities of 14 chronic diseases from 2015 to 2020.

		2015		2018			2020		
	Prevalence rate	Comorbidity rate^a^	x̄±s	Prevalence rate	Comorbidity rate	x̄±s	Prevalence rate	Comorbidity rate	x̄±s
**Hypertension**	27.1%	70.7%	2.471.43	35.8%	80.3%	3.011.71	42.6%	85.4%	3.502.00
**Dyslipidemia**	10.6%	86.5%	3.131.56	18.7%	91.0%	3.651.80	26.4%	93.7%	4.132.02
**Diabetes**	6.4%	84.3%	3.181.67	10.6%	90.5%	3.801.92	14.8%	94.4%	4.372.18
**Cancer**	0.6%	57.7%	2.692.19	1.4%	82.6%	3.412.30	2.2%	89.4%	4.082.48
**Chronic Lung Diseases**	7.2%	83.1%	3.051.66	10.6%	90.6%	3.781.95	15.3%	93.6%	4.432.24
**Liver Disease**	2.8%	84.0%	3.291.81	5.4%	90.0%	4.002.12	7.5%	93.2%	4.752.47
**Heart Attack**	10.7%	89.6%	3.371.58	16.2%	93.8%	3.921.83	20.8%	96.3%	4.512.08
**Stroke**	1.8%	87.2%	3.431.77	5.4%	94.0%	4.061.99	7.5%	96.3%	4.822.32
**Kidney disease**	4.5%	87.9%	3.411.81	7.4%	92.8%	4.052.12	10.3%	95.4%	4.782.38
**Stomach Disease**	20.1%	73.8%	2.531.42	26.9%	82.1%	3.081.75	31.4%	88.0%	3.672.07
**Emotional Problems**	1.0%	72.9%	3.102.14	1.5%	87.1%	4.002.50	4.1%	93.0%	4.962.71
**Memory-Related Disease**	1.1%	89.6%	3.611.94	2.6%	93.6%	4.522.20	5.7%	97.0%	5.392.41
**Arthritis**	29.2%	68.7%	2.381.40	35.7%	78.7%	2.941.73	40.9%	85.1%	3.502.04
**Asthma**	2.9%	94.2%	3.651.78	4.4%	97.9%	4.391.99	5.9%	99.0%	5.212.28

^a^: The proportion of individuals with a specific disease who also have one or more additional diseases. Comorbidity rate = number of individuals with the disease who have at least one other disease/total number of individuals with that disease.

### 3.3 Combination of chronic diseases

In this study, the Apriori algorithm was used to identify association rules between chronic diseases [18], aiming to reflect the nature and strength of association among 14 chronic diseases in middle-aged and elderly populations. This analytical approach facilitated the exploration of prevalent comorbidity patterns and their evolution over time, providing empirical evidence to prioritize prevention and management strategies for geriatric chronic diseases comorbidities. In this study, we established a minimum support threshold of 3%, a minimum confidence level of 50%, and a lift greater than 1.5, with the final association rules ranked in descending order of lift.

The lift quantifies directional associations between antecedent and consequent events. It is generally assumed that a lift of 1 indicates statistical independence between the antecedent and the consequent, a lift below 1 suggests negative associations, and a lift exceeding 1 demonstrates positive associations, with magnitude reflecting correlation strength. However, there is no unified norm for setting the lift. While most studies adopt a lift of >1 as a baseline criterion [19–21], emerging evidence suggests that marginally elevated lift (approaching 1) may indicate weak correlations, and the etiology may be relatively independent [22]. In similar studies, Cheng et al. [23] implemented lift >2, Xu et al. [24] and Liu et al. [25] established lift >1.5, whereas Guo et al. [17] adopted lift >1.6 for ternary comorbidity analyses. Drawing from these empirical observations, our study set a lift of >1.5 to balance sensitivity and specificity in detecting significant comorbidity combinations.

**Fig 2:**
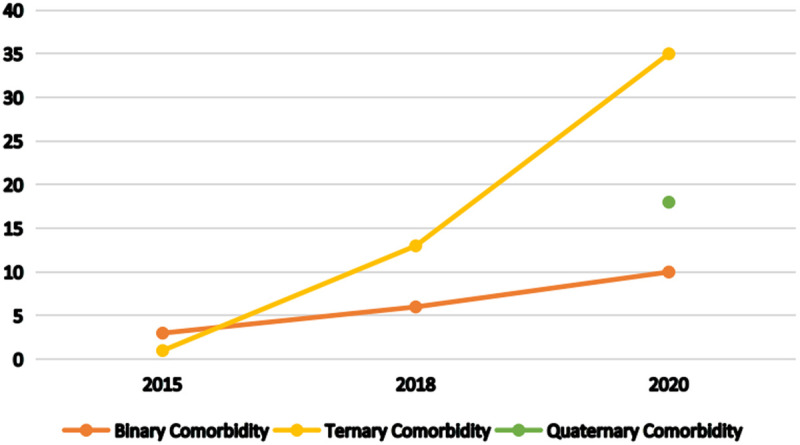
Variations in comorbidity combinations in China from 2015 to 2020.

Based on the aforementioned screening criteria, we found that the number of association rules identified demonstrated substantial growth; the number of strong association rules increased remarkably from four in 2015–63 in 2020. The key analytical advantage of line graphs (Fig 2) in temporal dynamic comparison is that they can simultaneously compare the change in trajectories and trends of different comorbidity combinations. The time-series presentation reveals that binary comorbidity combinations exhibited a steady upward trajectory with relatively stable growth patterns, whereas ternary comorbidity experienced an exponential surge, escalating from one to 35 cases during the observation period. Notably, quaternary comorbidity emerged for the first time in 2020, reaching 18 cases in their initial occurrence.

**Fig 3:**
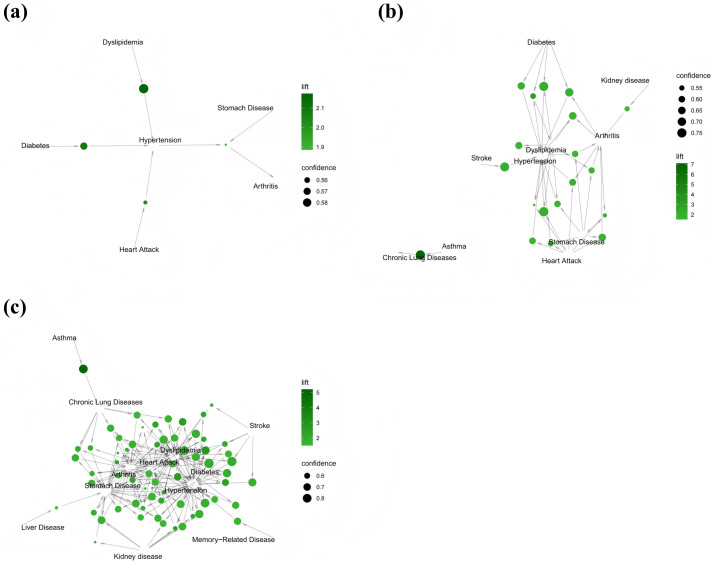
Network of comorbidity association rules in 2015-2020. (a: Network of comorbidity association rules in 2015; b: Network of comorbidity association rules in 2018; c: Network of comorbidity association rules in 2020).

Concurrently, we sequentially performed visualization of comorbidity association rules for the years 2015, 2018, and 2020. As illustrated in Fig 3, the graphical representation follows three distinct visual encodings: (1) node size corresponds proportionally to confidence levels, with larger diameters indicating higher confidence; (2) color intensity reflects lift, where deeper hues denote stronger lift values; and (3) directed arrows signify relationships between item sets, with the quantity of arrows at node junctions representing the count of comorbidities.

In 2015, screening identified three strong binary comorbidity rules and one ternary comorbidity rule (Fig 3a). Among binary comorbidity combinations, the highest lift was observed for “Hypertension + Dyslipidemia” (n = 631, lift = 2.17), followed by “Hypertension + Diabetes” (n = 369, lift = 2.10) and “Heart Attack + Hypertension” (n = 596, lift = 2.04). The most significant ternary association emerged as “Hypertension + Stomach Disease + Arthritis” (n = 332, lift = 1.89).

By 2018, the number of identified rules increased substantially to six binary and 13 ternary rules (Fig 3b). The binary combinations demonstrating superior association strength included “Asthma + Chronic Lung Diseases” (n = 331, lift = 7.10), “Stroke + Hypertension” (n = 402, lift = 2.05), and “Diabetes + Hypertension” (n = 680, lift = 1.78). In addition, the ternary comorbidity combination “Hypertension + Diabetes + Dyslipidemia” achieved the highest lift (n = 384, lift = 3.02). Of particular significance, the combination of asthma and chronic lung diseases exhibited independence from other associations, whereas interconnections among cardiovascular disorders became more pronounced compared to 2015 baseline observations.

The 2020 data demonstrated accelerated multimorbidity complexity, with 10 binary, 35 ternary, and 18 quaternary association rules identified (Fig 3c). Dominant binary associations included “Asthma + Chronic Lung Diseases” (n = 477, lift = 5.21), “Diabetes + Dyslipidemia” (n = 864, lift = 2.20), and “Stroke + Dyslipidemia” (n = 387, lift = 1.94). Among ternary comorbidity combinations, “Hypertension + Heart Attack + Dyslipidemia” showed the strongest association (n = 390, lift = 2.65), with the previously observed “Hypertension + Diabetes + Dyslipidemia” combination showing an enhanced lift value of 2.49 in 2020, marking a significant rise compared to its value in 2018. The emergent quaternary comorbidity profile was dominated by “Hypertension + Diabetes + Heart Attack + Dyslipidemia” (n = 332, lift = 2.84).

### 3.4 Pattern of chronic disease comorbidity

**Fig 4:**
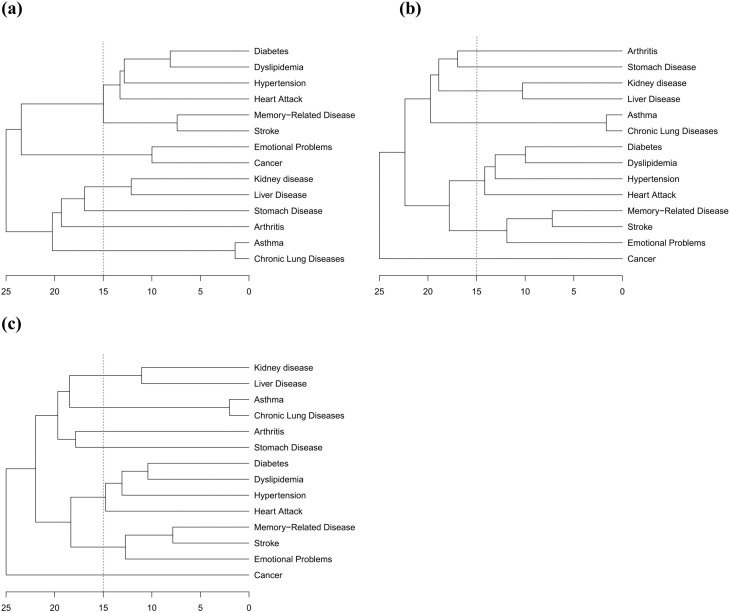
Tree cluster of comorbidities among middle-aged and elderly people in China in 2015, 2018, and 2020. (a: Tree cluster map of comorbidities of middle-aged and elderly people in China in 2015; b: Tree cluster map of comorbidities of middle-aged and elderly people in China in 2018; c: Tree cluster map of comorbidities of middle-aged and elderly people in China in 2020).

This study used Yule’s Q method [26], which assesses associations between categorical variables, to investigate comorbidity patterns in chronic diseases among middle-aged and elderly populations. Through logical distance calculations, we constructed cross-temporal dendrograms to visualize disease clustering dynamics. By systematically comparing clustering structures across three temporal cross-sections (2015, 2018, and 2020), this longitudinal analysis reveals the temporal evolution of comorbidity associations and characterizes developmental trajectories of multimorbidity patterns within China’s aging population (Fig 4).

The initial clustering structure in 2015 (Fig 4a) demonstrated that the associations of chronic diseases were driven by organ systems, comprising the following: respiratory comorbidity pattern (chronic lung disease and asthma); cardio-cerebral comorbidity pattern (stroke, memory-related disease, dyslipidemia, diabetes, hypertension, and heart attack); liver and kidney comorbidity pattern (liver disease and kidney disease); and cancer and emotional problems, alongside independent clusters for gastric disease and arthritis.

This broad organ system-based clustering underwent significant reorganization by 2018 (Fig 4b) and specifically manifested through the following: (1) differentiation of the cardio-cerebral comorbidity pattern into cerebrovascular comorbidity pattern (emotional problems, stroke, and memory-related diseases) and cardiovascular metabolic comorbidity pattern (dyslipidemia, diabetes, hypertension, and heart attack) and (2) dissociation of cancer from emotional problems comorbidity into an independent cluster. Consequently, the 2018 comorbidity network consolidated into four principal patterns: respiratory, liver and kidney, cerebrovascular, and cardiovascular metabolic comorbidity patterns.

By 2020 (Fig 4c), the comorbidity patterns stabilized with the completely same pattern compared to 2018, exhibiting only minor variations in merging distances. This consistency indicates completion of reorganization in disease association networks.

## 4 Discussion

This study analyzed the prevalence and evolution patterns of chronic diseases and comorbidities in middle-aged and elderly people aged ≥45 years in China using the CHARLS database from 2015–2020. The study revealed that the prevalence of chronic comorbidities among Chinese middle-aged and elderly individuals in 2020 was recorded at 59.9%. Notably, these figures were comparatively lower than the reported common prevalence of chronic comorbidities among middle-aged and elderly populations in Canada (66.7%), as documented by Nicholson et al. [27]. Wang et al. found that the more developed the regional economy is, the higher the prevalence of comorbidities [28]. Although China is still a developing country, the comorbidity of chronic diseases in middle-aged and elderly people is higher than that in other developed countries [29,30]. Furthermore, it is imperative to extensively address the prevailing comorbidity pattern in China that is characterized by cardiovascular metabolic comorbidity, which necessitates increased attention.

### 4.1 Chronic disease comorbidity is becoming more common

The prevalence of comorbidities in chronic diseases is increasingly prevalent among the middle-aged and elderly populations in China, as evidenced by a clear upward trend observed from 2015–2020. In 2015, the prevalence of chronic diseases in middle-aged and elderly people over 45 years old was only 34.3%; the prevalence reached 49.0% in 2018, and it was as high as 59.9% in 2020. This trend may be related to the respondents in the follow-up data used because an increase in age is one of the important factors affecting chronic disease comorbidity [31]. Furthermore, this phenomenon may be influenced by a multitude of additional factors, encompassing alterations in lifestyle and advancements in medical technology. Contemporary modifications in lifestyle, such as dietary changes, sedentary behavior, tobacco consumption, and alcohol misuse, create conducive circumstances for the emergence of chronic disease comorbidities [32]. In addition, advancements in medical technology have significantly enhanced the precision and efficacy of diagnosing and treating chronic diseases, thereby facilitating the identification of comorbidities [33].

### 4.2 The evolution of comorbidities in chronic diseases

In the analysis of association rules for chronic disease comorbidities, hypertension was most significantly associated with other cardiovascular and metabolic diseases and the changing trend of these association rules also showed an evolving trend. By exploring the physiological, environmental, and genetic factors underlying these association rules, we can gain a deeper understanding of the nature of chronic comorbidities and develop more efficacious strategies for prevention and treatment in the future.

In the association rule analysis conducted in 2015, four strongly associated comorbidity combinations (three binary comorbidity rules and one ternary comorbidity rule) were identified, and hypertension was consistently present in these combinations. This highlights the pivotal role of hypertension in the early-stage comorbidity network among middle-aged and elderly populations in China. Several studies have revealed that approximately 245 million Chinese adults are affected by hypertension [34]. As a precursor to multiple chronic health conditions, the presence of hypertension typically indicates elevated risks of developing comorbidities [35]. Elevated blood pressure induces progressive damage to multiple organ systems, including cardiovascular, cerebral, renal, and ocular systems, thereby exacerbating the overall disease burden in affected individuals. These evidences highlight the critical necessity for early detection and timely therapeutic intervention in hypertension management.

The number of combinations of chronic disease comorbidity significantly increased to 19 in 2018. While hypertension retained its central role in comorbidity networks (present in 14 combinations), dyslipidemia-associated comorbidities exhibited a marked surge, rising from one combination in 2015 to eight combinations in 2018, paralleling the growth trajectory of hypertension-related clusters. Additionally, arthritis-involved combinations increased to eight. Analysis of lift values revealed that the binary comorbidity “Chronic Lung Diseases + Asthma” demonstrated the strongest association strength, whereas the ternary comorbidity “Hypertension + Diabetes + Dyslipidemia” ranked highest in lift values. Pan et al. [22] reported that this ternary comorbidity exhibited a high incidence ratio and shared etiological pathways and synergistic risk factors.

By 2020, chronic comorbidity combinations escalated to 63, with hypertension remaining predominant (38 combinations). Dyslipidemia-associated comorbidities further expanded to 36, and synergistic relationships with hypertension were intensified. Previous studies have established dyslipidemia as both a risk factor and consequence of hypertension, indicating bidirectional causality and synergistic effects [36–38]. Arthritis-related combinations reached 31, warranting clinical attention despite being slightly lower than the former two. Notably, the binary comorbidity “Chronic Lung Disease + Asthma” maintained its highest lift value, while quaternary comorbidity patterns emerged for the first time, reflecting escalating complexity in disease interactions and multifactorial pathogenesis.

### 4.3 The evolution of chronic disease comorbidity patterns

Analysis of three-wave cross-sectional data (2015–2020) reveals the evolutionary trajectory of three stages in comorbidity patterns among Chinese middle-aged and elderly populations. The baseline phase (2015) exhibited an organ system-dominant clustering pattern. This configuration transitioned into a risk factor-driven paradigm in 2018, and this restructured network persisted with progressive consolidation through 2020, establishing a stable comorbidity architecture.

In 2015, chronic lung diseases and asthma had the strongest correlation among respiratory diseases, forming a distinct comorbidity pattern. In previous studies, the correlation between the two has been confirmed several times, and asthma and chronic lung diseases exhibit significant similarities in terms of etiology and influencing factors [39]. Liver disease and kidney disease are grouped into one cluster, forming a pattern of liver and kidney comorbidity. The correlation between the two has also been reflected in previous studies [40,41]; however, the etiological relationship between the two is relatively independent [22]. For instance, severe liver disease may be accompanied by renal insufficiency, whereas liver disease does not induce kidney disease under normal circumstances [42]. The relationship between gastric disease and arthritis has been mentioned in many studies [43,44]; however, the two diseases did not form clusters in this study. Cancer, which clustered together with emotional problems, is caused by the uncontrolled proliferation of abnormal cells. It is challenging to treat, and patients are likely to experience negative emotions after diagnosis, which may be due to the disease itself, demographic factors, or physical changes and stigma [45]. Studies have shown that the incidence of mental pain in cancer patients in China is 24.2%–73.4% [46], and the probability of cancer patients suffering from depression and anxiety is very high. These suggest that cancers are closely related to emotional and mental problems. The cardio-cerebral comorbidity pattern, identified in 2015, includes a wide range of chronic diseases, and the heart and brain are genetically, structurally, and functionally related [47]. The shared etiology of cardio-cerebral comorbidity lies in the pathogenesis of atherosclerosis—with hypertension, hyperlipidemia, and hyperglycemia being risk factors for atherosclerosis [48]—which can easily predispose individuals to coronary artery disease, stroke, and other related disorders.

By 2018, the metabolic risk factor-driven cerebrovascular comorbidity pattern and cardiovascular metabolic comorbidity pattern had replaced the cardio-cerebral comorbidity pattern observed in 2015. During this period, the association between chronic pulmonary disease and asthma remained the most robust comorbidity combination, clustering the earliest. Meanwhile, cancer transitioned from co-clustering with emotional problems to forming distinct independent clusters. Psychological interventions for cancer patients, including cognitive behavioral therapy, mindfulness-based interventions, and relaxation techniques, have been increasingly implemented in clinical practice in recent years [49]. This progress has contributed to a reduction in the prevalence rates of psychiatric comorbidities such as depression and anxiety among cancer populations compared to the baseline, and the distinct pathophysiological origins of cancers, which differ fundamentally from other chronic diseases, classify them as an independent cluster. The cerebrovascular comorbidity pattern encompasses emotional problems, stroke, and memory-related diseases. Patients with stroke frequently manifest cognitive sequelae, memory impairment, and sensory deficits, whereas psychiatric disorders often involve cognitive dysfunction, behavioral abnormalities, and a decline in daily functioning [50–52]. The cardiovascular metabolic comorbidity pattern is considered to be the most replicable comorbidity pattern, although specific disease composition within distinct databases may introduce minor variations [53]. For instance, the incidence of dyslipidemia was not considered in the CKB project [54]. Mechanistically, hyperlipidemia can easily lead to arteriosclerosis, blood vessel stenosis, and blood pressure rise, thereby elevating the risk of hypertension [55]. Concurrently, chronic hyperglycemia can trigger endothelial damage, accelerating atherosclerotic progression, while hypertension can also lead to abnormalities in vascular structure and function, aggravate disorders of systemic vascular function, and then accelerate the evolution of diabetic complications. Hypertension, diabetes, and dyslipidemia are often considered risk factors for heart disease [56].

By 2020, comorbidity patterns remained structurally consistent with the observations in 2018. However, correlated with demographic shifts, the chronic disease prevalence has also increased. Compared to earlier clusters in 2015 and 2018, the inter-disease dissimilarities in 2020 decreased, the disease prevalence profiles converged, and the comorbidity interactions within the aging population stabilized.

## 5 Conclusions

This study conducted an in-depth exploration of the comorbidity of chronic diseases among middle-aged and elderly people in China and revealed that the prevalence and comorbidity rates of chronic diseases have been increasing annually. The problem of comorbidities in chronic diseases among Chinese middle-aged and elderly people is prominent and serious. Meanwhile, the number of strong association rules has grown rapidly, with hypertension topping the list in terms of associated rules and cardiovascular metabolic diseases showing a high degree of correlation. Our study also revealed similarities and differences in comorbidity modes derived from three years of follow-up data and revealed a trend of convergence in these modes, providing important insights for further understanding and preventing comorbidities of chronic disease among middle-aged and elderly people. To actively address the problem of comorbidities among middle-aged and elderly people in China and promote healthy aging, the author suggests focusing on key diseases such as hypertension and arthritis as breakthrough points. Moreover, attention should be given to the differences in comorbidities of chronic diseases among middle-aged and elderly people across different sexes and age groups. Guidelines for the prevention and treatment of chronic disease comorbidities should be established, providing basic principles for risk factor control, assessment, prevention, and treatment of chronic disease comorbidities.

Our study has certain limitations. Although individuals who passed away during the study period or those who failed to complete all three follow-ups were excluded to obtain a more homogeneous study population, this may have led to a misjudgment of the impact of high-mortality diseases.
